# Context-dependent interactive effects of non-lethal predation on larvae impact adult longevity and body composition

**DOI:** 10.1371/journal.pone.0192104

**Published:** 2018-02-05

**Authors:** Karthikeyan Chandrasegaran, Samyuktha Rao Kandregula, Suhel Quader, Steven A. Juliano

**Affiliations:** 1 National Centre for Biological Sciences, Tata Institute of Fundamental Research, Bengaluru, Karnataka, India; 2 School of Biological Sciences, Illinois State University, Normal, Illinois, United States of America; 3 SASTRA University, Tirumalaisamudram, Thanjavur, Tamil Nadu, India; 4 Centre for Ecological Sciences, Indian Institute of Science, Bengaluru, Karnataka, India; 5 Nature Conservation Foundation, Mysuru, Karnataka, India; University of Vienna, AUSTRIA

## Abstract

Predation impacts development, behavior and morphology of prey species thereby shaping their abundances, distribution and community structure. Non-lethal threat of predation, specifically, can have a strong influence on prey lifehistory characteristics. While investigations often focus on the impact of predation threat on prey in isolation, tests of its interactive effects with food availability and resource competition on prey survival and fitness can improve understanding of costs, benefits and trade-offs of anti-predator strategies. This study, involving *Aedes aegypti* mosquitoes as a model organism, investigates both simple and interactive effects of predation threat during the larval stage on survival, size at and time to maturity, stored teneral reserves of glycogen, protein and lipid in adults, and adult longevity. Our results show that development times of mosquito larvae were increased (by 14.84% in males and by 97.63% in females), and size of eclosing adults decreased (by 62.30% in males and by 58.33% in females) when exposed to lowered nutrition and elevated intraspecific competition, but that predation had no detectable effect on these simple traits. Teneral reserves of glycogen, protein and lipid and adult longevity were positively correlated with adult body size. Non-lethal predation threat had significant interactive effects with nutrition and larval competition on teneral reserves in males and adult longevity in males and females. The sexes responded differently to conditions encountered as larvae, with the larval environment affecting development and adult characteristics more acutely for females than for males. The outcome of this study shows how threat of predation on juveniles can have long-lasting effects on adults that are likely to impact mosquito population dynamics and that may impact disease transmission.

## Introduction

Ecology plays a crucial role in shaping life history characteristics. Both biotic (e.g., predators, competitors, parasites) and abiotic (e.g., temperature, humidity) components of the environment drive adaptations in organisms, resulting in environment-specific phenotypes [1–4]. Predation in particular has major effects on fitness of prey by impacting their survival, growth and reproduction [5–8]. These fitness effects select for individuals that invest in adaptive plastic traits that enable them to avoid predation [9,10]. Predation impacts prey organisms via both lethal or non-lethal effects [11–13]. While the lethal effect of predation results in prey mortality, non-lethal effects of predation trigger a suite of morphological, physiological and behavioral responses in prey [14–17]. Both lethal and non-lethal effects of predation can affect prey distribution and abundance and prey population dynamics, thus producing long-term evolutionary changes in animal communities [5,18,19].

Morphological and physiological responses to non-lethal predation threat are evident in aquatic organisms with complex life cycles [10,13,15,20,21]. For such organisms, metamorphosis may be an opportunity to escape predators of the larval habitat. Well-studied examples occur among anuran tadpoles. When exposed to risk of predation, tadpoles exhibit altered body shape and reduced development time [22,23]. While altered body shape can enhance swimming speed of tadpoles, faster development facilitates escape from the threat-laden environment [24–26]. However, these defense strategies have their own costs to prey. Greater development rate reduces the time spent feeding and growing by tadpoles, resulting in anurans that are smaller at metamorphosis, thus impacting survival and overall fitness [27,28]. A broad range of other environmental effects (e.g., food availability, competition, disease) also influence lifehistory traits [29–32]. Altering development rates or responding to enemies in a nutrition-limited or crowded environment are likely to be physiologically more challenging than in a nutrition-abundant environment devoid of competition. For instance, organisms metamorphosing in crowded or nutrient-limiting larval environments have slower growth and development rates and emerge as smaller adults [18,33–35] that are expected to suffer survival and reproductive costs. Slow growth rates can be a consequence of reduced feeding and selection of low-food habitats low in predation risk, and such habitat selection can increase inter- and intra-specific competition [36–39]. Because predation can affect so many aspects of organisms’ lives it seems likely that predation will interact with other ecological factors, making the optimal phenotype context dependent [5,40–42]. In that case, the costs of evading predation under different conditions are expected to be different and their influence on adult characteristics is likely context dependent [43,44]. Despite this expectation of multifaceted and context dependent effects of threat of predation, most studies investigating effects of predation threat on prey focus on behavioral effects and more rarely on prey traits, typically development time, adult size, and survival, in isolation [5,8,45]. Testing for multifaceted, interactive effects of predation and other ecological factors could provide a more complete understanding of the costs, benefits, and trade-offs of antipredator responses. Because we expect responses to predation threat to be costly, the most interesting potentially interacting factors are those that are related to the organism’s ability to pay those costs: food availability and population density.

We use a model system of mosquitoes: developing *Aedes aegypti* larvae exposed to threat of predation from *Toxorhynchites rutilus* larvae. Mosquitoes have short but complex life cycles, rendering them an ideal model system for this study [46,47]. Mosquito larvae often show behavioral changes on exposure to chemical cues from predation [48–56]. As is true for most aquatic systems, these chemical cues originate either from injured prey or directly from the predator [57–60]. Effects of predation threat on larval growth and adult traits of mosquitoes have been infrequently examined [61–64]. Two-way interactions between nutrition availability, competition for resources and larval predation affect development and survival of mosquito larvae [61,65]. These effects on larvae could subsequently influence adult physiological and demographic traits (e.g., energy reserves, size, longevity), and because mosquitoes like *A*. *aegypti* are vectors of human pathogens, these effects may impact the ability of the adult mosquito to act as a vector [39,66–68].

The aim of this study is to test the hypothesis that nutrition availability and intraspecific competition for resources interact with non-lethal predation threat to shape life history. We specifically postulate that impacts of predation threat on adult traits will be more severe when food is scarce or population density is high. We address this objective using a factorial experiment with three levels of nutrition, two levels of conspecific larval density, and three levels of non-lethal exposure to predator cues. We analyzed how survival to adulthood, development rate, adult body size, teneral reserves (glycogen, protein, lipid), and adult longevity were impacted by these factors. We employ methods derived from existing protocols [69–71] to quantify stored glycogen, lipid and protein as indicators of adult lifehistory traits and to learn how complex interactive effects of the larval environmental drive investments in adult body size and nutritional reserves, and ultimately longevity.

## Materials and methods

Eggs of the yellow fever mosquito (*Aedes aegypti*) from a colony originating from New Orleans LA, USA, were hatched in deionized (DI) water 24 hours prior to the start of the experiment. A stock suspension of larval nutrient medium was prepared by adding 1g of freshly powdered mixture of dog biscuit (Ol’ Roy, Doane Pet food, Brentwood TN, USA: 24% protein, 11% lipid, 57% carbohydrate by dry weight) and yeast (3:2) per 300ml of DI water. The nutrient medium was then incubated at 25°C for 24 hours, then passed through a 1.5 mm mesh to remove large particulates, and dilutions of 30%, 15% and 10% in DI water were used as high, medium and low nutrition treatments, respectively.

First instar larvae were randomly added to experimental units (0.5 L plastic containers 11.94 cm in diameter and 7.37cm tall, filled with 300 ml of larval nutrient medium; see below) assigned a combination of 3 nutrition treatments, 3 predator treatments and 2 levels of competition. In each experimental unit, a PVC pipe enclosure 3.5 cm in diameter containing the predator treatment was immersed into the nutrient medium. The submerged end of the pipe was sealed with two layers of nylon mesh (0.6 mm and 0.3 mm) on the inside and outside, respectively. The mesh ensured that nutrient medium passed freely in and out of the pipe but prevented mosquito larvae from entering the predator enclosure. The outer 0.3 mm mesh was removed after all larvae in the container reached the third instar.

Two levels of larval competition (low and high) consisted of initial densities of 26 and 78 first instar larvae per experimental container. The 3 predator treatments were control, simulated predation, and live predation. For live predation, a 3^rd^ or 4^th^ instar *T*. *rutilus* larva was added to the PVC enclosure and offered 10 fourth instar *A*. *aegypti* larvae daily. For simulated predation, fourth instar *A*. *aegypti* larvae were crushed with forceps and added to the PVC enclosure daily. The number of crushed *A*. *aegypti* larvae per enclosure was the mean number of *A*. *aegypti* larvae eaten by the *T*. *rutilus* larvae across the live predator treatment. In controls, nothing was added into the PVC enclosure. Two replicate containers were assigned per treatment combination and this experiment (referred to as a ‘block’) was repeated thrice, thus bringing the total of number of individual containers (experimental units) to 108 (= 2 densities x 3 predation treatments x 3 nutrition levels x 6 replicate containers). Experimental containers were housed in an incubator at 25°C and 80% RH with a 14:10 h day-night cycle. Mosquito larvae were monitored daily at 0000, 0600, 1200 and 1800 hours until adult eclosion. Pupae were transferred to individually labelled, mesh-covered glass vials. On eclosion, sex was determined and wet adult body mass (±1.0 μg) was determined for every second individual using a Cahn C31 microbalance. Post weighing, individuals were flash frozen in liquid nitrogen and stored at -80°C for assays of stored reserves of glycogen, protein and lipids (see below). Remaining adults were kept alive in individually labelled glass vials, provided with only water, and survival was monitored every 2 hours until all had died. Post death, wings were dissected and lengths (from anal lobe to wing tip) were measured to the nearest 0.1 mm.

### Biochemical assays

#### Sample preparation

A frozen mosquito was thawed on ice prior to homogenization in 50 μl of 2% sodium sulfate solution in a 2.0 ml centrifuge tube, and 450μl of chloroform-methanol (1:2 v/v) solution was then added to the homogenized sample. Aliquots of 275μl, 75 μl, and 150 μl of the homogenate were set aside for glycogen, protein and lipid assays, respectively. The assays described below were adapted from [71,72].

#### Glycogen estimation

The homogenate (275μl) was vortexed and centrifuged for 2 minutes at 13000 rpm and 4°C. The supernatant was discarded and the pellet was re-suspended in 100μl of 2% sodium sulfate solution. Care was taken to ensure the pellet disintegrated completely. In case of very small/loose pellets, the resuspension was heated at 90°C to let the supernatant evaporate until it was reduced to 100μl. Following this, 100μl of 2% sodium sulfate solution was added and the mixture was heated at 90°C in a dry bath to evaporate remaining solvent. The dried pellet was then cooled on ice and 1 ml of anthrone was added. The mixture was vortexed until the pellet completely disintegrated and the mixture was incubated in a 90°C water bath for 90 seconds. The mixture was cooled on ice to stop the reaction and absorbance was measured at 625 nm using D-glucose as the standard. This and all other spectrophotometric assays were done on a 96-well plate reader (3370; Corning™, USA)

#### Protein estimation

The homogenate (75μl) was mixed with 600μl of Thermo Scientific™ Micro BCA™ protein assay reagent (23225; Thermo Scientific™, USA) and vortexed for 30 seconds. The mixture was incubated at room temperature for 2 hours and absorbance was measured at 562 nm using a dilution-series of BSA (611910100; Thermo Scientific™, USA) as standard. The end product is stable for 15–20 minutes.

#### Lipid estimation

The homogenate (150μl) was heated at 90°C in a dry bath until complete evaporation of the solvent. The pellet was then cooled on ice, mixed with 20 μl of sulfuric acid, heated in a 90°C water bath for 2 minutes and cooled on ice. Vanillin reagent (480 μl) was added to the mixture and vortexed. After incubation for 10 minutes at room temperature, absorbance was measured at 525 nm using dilutions of commercial vegetable oil in chloroform as a standard. The end product is stable for 30 minutes.

### Statistical analysis

In all the analyses except survival to adulthood we analyze responses of males and females separately, because of the well-known sexual dimorphism in development time, body size, and adult physiology [73–75]. We thus expect different effects of manipulated variables on the sexes. This separation also limits the design to only 3 manipulated factors plus their interactions. MANOVA was used to test for effects of nutrition, intraspecific competition and predation treatments on development time and adult body mass. MANCOVA yielded tests for effects of three independent variables on stored nutrient reserves with adult body mass as a covariate. Given the positive correlation between adult body size and longevity, Cox’s proportional hazard model was used to analyze adult longevity with wing length as a covariate to estimate the hazard of death [76]. ANOVA was used to analyze proportion of larvae surviving to adulthood across treatment categories. All variables including any covariates were log-transformed in MANCOVA and survival analysis. All the above analyses were performed using mixed model with the three independent variables being treated as fixed effects and individual container nested within block-competition-nutrition-predation combination being treated as random effect. This last random effect represents random variation among individual experimental units receiving the same treatment combinations. It is thus appropriate as the error term for analysis of effects of manipulated variables and interactions. The assumptions of normality, homogeneity of variances, and linearity were met in all analyses, and the assumption of homogeneity of slopes relative to the covariate was tested whenever required using interactions of experimental factors and covariates. When slopes did not differ significantly, those interactions were omitted from analysis resulting in ANCOVA or MANCOVA. When the slopes were significantly inhomogeneous, we focus further testing on the differences among treatment groups in slopes relative to the covariate. For MANOVA and MANCOVA we used *F* statistics derived from Pillai’s Trace [77,78]. All significant MANOVA and MANCOVA effects were followed up with multivariate pairwise contrasts [78], with sequential Bonferroni correction for multiple tests within each MANOVA and MANCOVA at experimentwise α = 0.05 [79]. The contributions of individual dependent variables to significant multivariate effects were interpreted using standardized canonical coefficients [78]. SAS PROC GLM was used for all MANOVAs; SAS PROC PHREG was used for longevity analyses; SAS PROC GLIMMIX was used for analysis of survival to adulthood [77].

## Results

### Larva–adult transition

**Fig 1:**
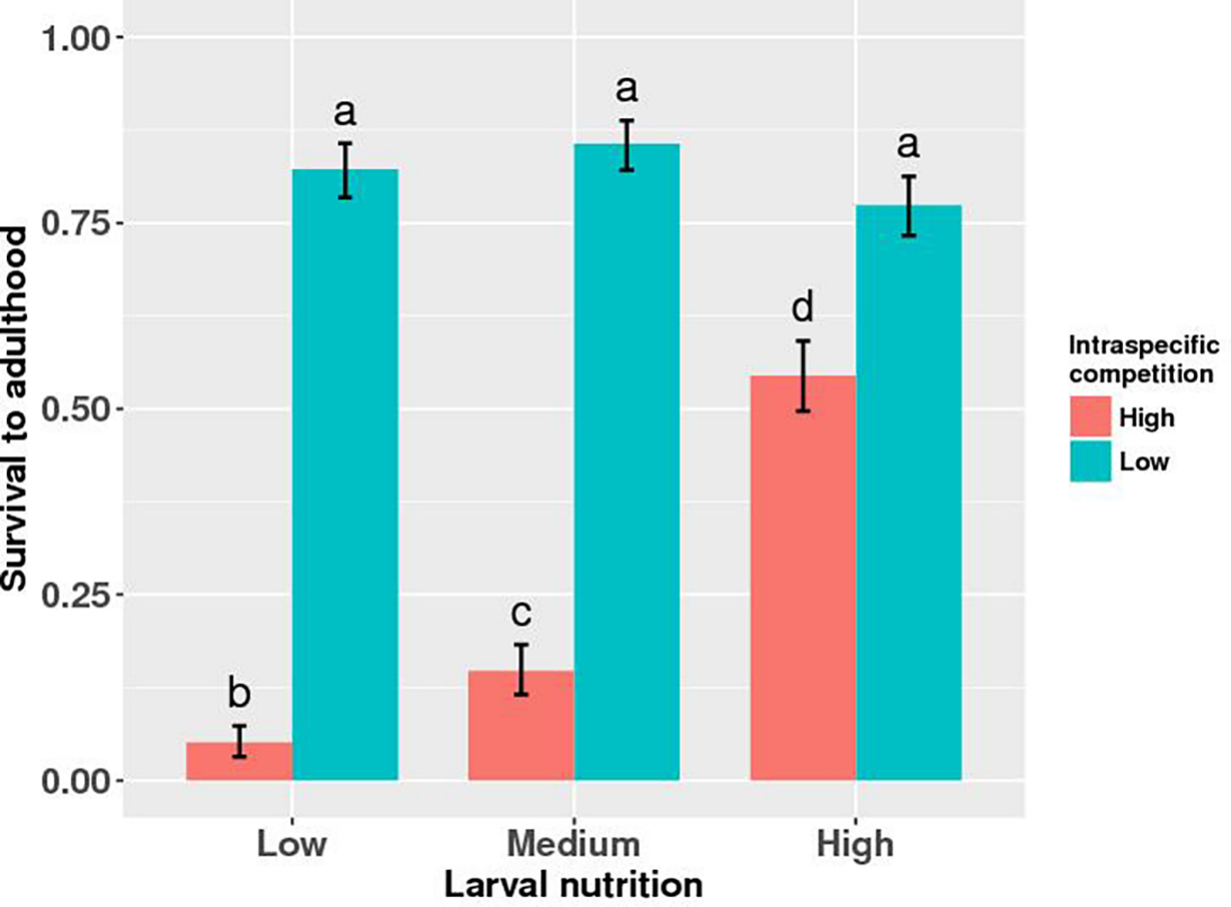
Survival to adulthood. Proportion survivorship to eclosion of *Aedes aegypti* larvae exposed to intraspecific competition and nutrition treatments. Values plotted are least squares means ± SE pooled across three predation treatments. Data were statistically tested using ANOVA and significant effects are described in Table 1. Treatment means associated with the same letters are not significantly different.

*Aedes aegypti* survivorship to adulthood was significantly affected by intraspecific competition, nutrition availability, and their interaction, but not by predation treatment, or any of its interactions (Table 1). Proportion of larvae surviving to adulthood decreased significantly with decreasing nutrition levels at high larval density but was unaffected by varying nutrition availability at low larval density (Fig 1 and Table 1).

**Table 1:** Survival to adulthood.

Source	*df*	F value	*P*
Competition	**1,88**	**471.02**	**<0.0001**
Nutrition	**2,88**	**28.04**	**<0.0001**
Predation	2,88	0.74	0.4808
Comp. × Nut.	**2,88**	**46.70**	**<0.0001**
Comp. × Pred.	2,88	0.76	0.4695
Nut. × Pred.	4,88	0.62	0.6516
Comp. × Nut. × Pred.	4,88	0.23	0.9222

ANOVA results for proportion of *Aedes aegypti* larvae surviving to adulthood (data pooled across both the sexes, arcsine (square root) transformed). Significant main effects and interactions are indicated in boldface. Pairwise contrasts between treatment categories are not included for brevity.

### Females

**Fig 2:**
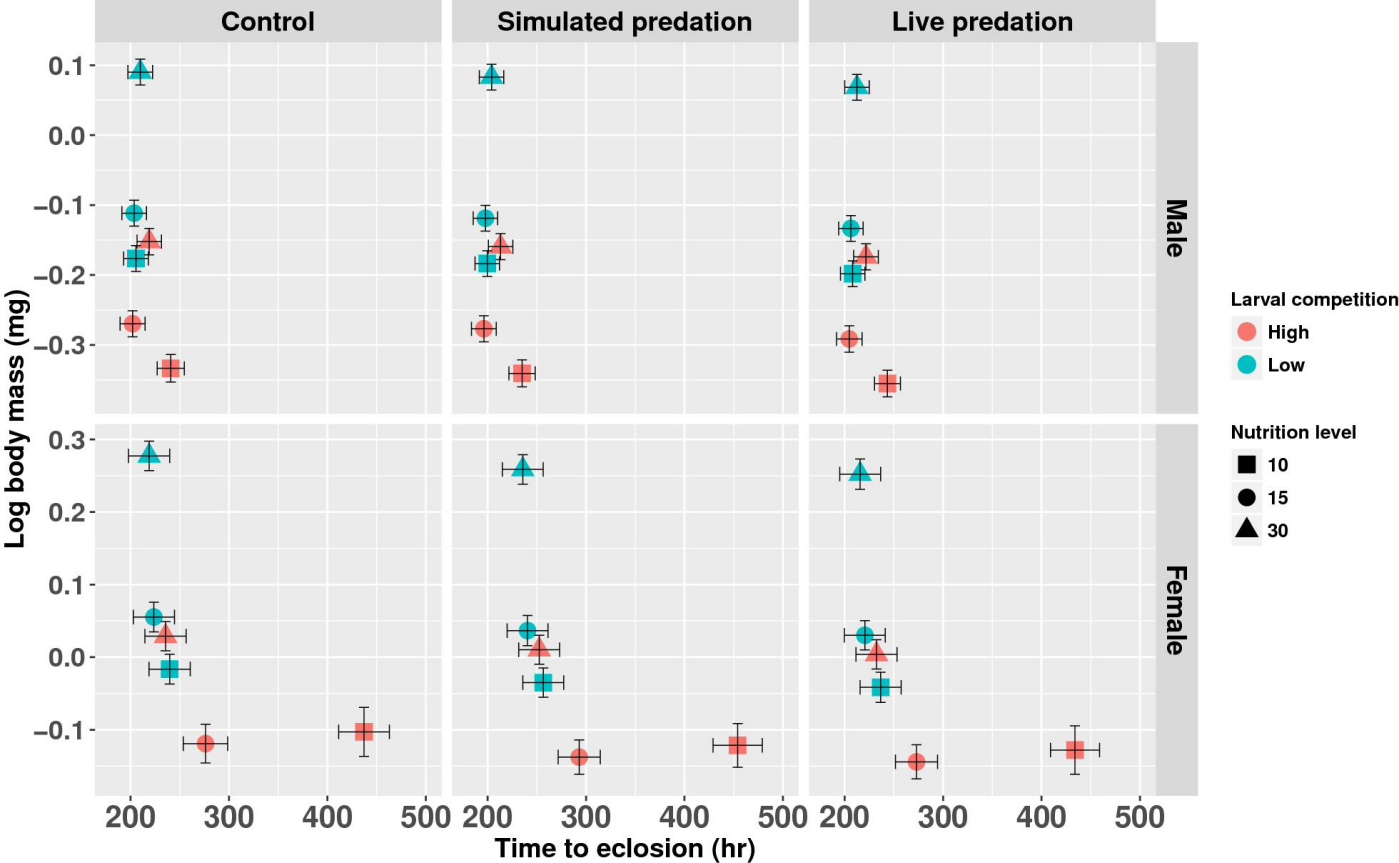
Time to eclosion and adult body mass. Bivariate plot of least squares means (±SE) for mean adult body mass and mean of median time to adult eclosion for larval nutrition × intraspecific competition across replicate containers. Data have been presented separately for male and female mosquitoes across three predation treatments. Data were statistically tested using a mixed model MANOVA and significant effects are described in Table 2.

MANOVA yielded significant effects of larval competition, nutrition availability and their interaction on development time and body mass of female mosquitoes (Fig 2 and Table 2). None of the main effects or interactions involving predation treatment was significant (Table 2). Multivariate pairwise contrasts revealed significant differences for nine of the pairwise comparisons for effects of nutrition and competition (Table 2). Standardized canonical coefficients (SCC) for time to eclosion and adult body mass were of opposite signs for most significant effects, indicating that larval competition and nutrition treatments that decreased time to eclosion also increased adult body mass (Table 2). Increased nutrition and decreased intraspecific competition resulted individuals eclosing significantly earlier at significantly greater adult weight. For females, SCCs for competition-nutrition interaction were both positive indicating that some combinations yielded positive associations of female development time and adult size (See Fig 2, compare means for females at high competition-low nutrition to females at high competition-medium nutrition).

**Table 2:** Time to eclosion and adult body mass.

Source	MALE					FEMALE				
	Pillai’s trace (*F*)	*df*	*P*	Standardized canonical coefficients		Pillai’s trace (*F*)	*df*	*P*	Standardized canonical coefficients	
				Time to eclosion	Adult body mass				Time to eclosion	Adult body mass
Competition	**0.8456**	**2,65**	**<0.0001**	**-0.2550**	**3.3886**	**0.7203**	**2,50**	**<0.0001**	**-0.6267**	**2.4183**
Nutrition	**0.8837**	**4,132**	**<0.0001**	**-0.2033**	**3.3954**	**1.1060**	**4,102**	**<0.0001**	**-0.4407**	**2.5719**
									**1.2982**	**0.8553**
Predation	0.0563	4,132	0.4339			0.0833	4,102	0.3569		
Comp. × Nut.	**0.4982**	**4.132**	**<0.0001**	**-0.1556**	**3.3926**	**0.6846**	**4,102**	**<0.0001**	**0.8545**	**2.1090**
Comp. H vs. L in Nut. L	**0.4605**	**2,65**	**<0.0001**	**-0.3930**	**3.3162**	**0.4719**	**2,50**	**<0.0001**	**1.3385**	**-0.6033**
Comp. H vs. L in Nut. M	**0.5068**	**2,65**	**<0.0001**	**-0.1926**	**3.3955**	**0.3817**	**2,50**	**<0.0001**	**-0.2272**	**2.6756**
Comp. H vs. L in Nut. H	**0.8209**	**2,65**	**<0.0001**	**-0.2222**	**3.3941**	**0.7920**	**2,50**	**<0.0001**	**-0.0460**	**2.7093**
Nut. L vs. M in Comp. L	**0.1864**	**2,65**	**0.0012**	**-0.2214**	**3.3942**	**0.1831**	**2, 50**	**0.0064**	**-0.3257**	**2.6367**
Nut. L vs. H in Comp. L	**0.8436**	**2,65**	**<0.0001**	**-0.1858**	**3.3954**	**0.8241**	**2, 50**	**<0.0001**	**-0.0688**	**2.7077**
Nut. M vs. H in Comp. L	**0.7727**	**2,65**	**<0.0001**	**-0.1765**	**3.3949**	**0.7496**	**2, 50**	**<0.0001**	**-0.0019**	**2.7103**
Nut. L vs. M in Comp. H	**0.1518**	**2,65**	**0.0047**	**-0.6403**	**2.9404**	**0.3988**	**2, 50**	**<0.0001**	**1.3554**	**0.3897**
Nut. L vs. H in Comp. H	**0.5226**	**2,65**	**<0.0001**	**-0.2968**	**3.3752**	**0.5101**	**2, 50**	**<0.0001**	**1.2831**	**3.3752**
Nut. M vs. H in Comp. H	**0.3244**	**2,65**	**<0.0001**	**-0.0582**	**3.3606**	**0.2698**	**2,50**	**0.0004**	**-0.1739**	**2.6905**
Comp. × Pred.	0.0495	4,132	0.5038			0.0471	4,102	0.6532		
Nut. × Pred.	0.0429	8,132	0.9390			0.0733	8,102	0.8642		
Comp. × Nut. × Pred.	0.0547	8,132	0.8796			0.2073	8,102	0.1757		

MANOVA results and standardized canonical coefficients for time to eclosion and adult body mass of male and female *Aedes aegypti* mosquitoes. For significant main effects and interactions, multivariate pairwise contrasts of factor levels are included. Significant effects in MANOVA (significant pairwise comparaisons at experimentwise α = 0.05; sequential Bonferroni correction) are highlighted in boldface. For significant effects, standardised canonical coefficients for the first and second (if any) canonical variates are included; the magnitude of standardised canonical coefficients indicate the magnitude of the variable’s contribution to the significant effects in MANOVA (Scheiner, 1993).

MANCOVA showed that none of the main effects or interactions significantly affected teneral reserve components glycogen, protein and lipid after scaling these components to body mass, which had a significant effect on reserve components (Table 3). Bigger mosquitoes had proportionally greater teneral reserves. Standardized canonical coefficients indicated that lipid contributed the most and glycogen the least to the observed relationship between reserve components and adult body mass. The absence of any significant effect of interactions between independent variables and body mass implies that composition of reserve components is strongly and consistently related to adult body mass for females (Slopes ± SE for each of three teneral reserves vs body mass: Glycogen, 0.6511±0.067; Protein, 0.7568±0.078; Lipid, 0.6647±0.044).

**Table 3:** Stored teneral reserves.

Source	MALE						FEMALE					
	Pillai’s trace (*F*)	*df*	*P*	Standardized canonical coefficients			Pillai’s trace (*F*)	*df*	*P*	Standardized canonical coefficients		
				Glycogen	Protein	Lipid				Glycogen	Protein	Lipid
Body mass	**0.2987**	**3,461**	**< 0.0001**	**0.1880**	**0.6688**	**1.1385**	**0.4760**	**3,313**	**<0.0001**	**0.1226**	**0.5064**	**1.4514**
Competition	0.0171	3,64	0.7735				0.0557	3,49	0.4179			
Nutrition	0.1248	6,130	0.2033				0.2240	6,100	0.0596			
Predation	0.1639	6,130	0.0801				0.0703	6,100	0.7238			
Comp. × Nut.	0.1243	6,130	0.2057				0.1606	6,100	0.2015			
Comp. × Pred.	0.1554	6,130	0.0990				0.1451	6,100	0.2625			
Nut. × Pred.	**0.3302**	**12,198**	**0.0226**	**-4.2610**	**4.0306**	**1.4779**	0.3202	12,153	0.1211			
Comp. × Nut. × Pred.	0.2948	12,198	0.0505				0.2045	12,153	0.5159			
B.mass × Comp.	**0.0462**	**3,461**	**<0.0001**	**5.4899**	**-4.9868**	**0.6345**						
B.mass × Nut.	**0.0798**	**6,924**	**<0.0001**	**5.4174**	**-5.1631**	**0.7946**						
B.mass × Pred.	**0.0457**	**6,924**	**0.0015**	**3.7711**	**-2.9511**	**0.8267**						
B.mass × Comp. × Nut.	**0.0353**	**6,924**	**0.0114**	**3.4129**	**-2.2055**	**-0.0941**						
B.mass × Comp. × Pred.	0.0113	6,924	0.5137									
B.mass × Nut. × Pred.	0.0304	12,1389	0.2886									
B.mass × Comp. × Nut. × Pred.	**0.0807**	**12,1389**	**0.0002**	**-1.9586**	**1.9082**	**1.5171**						

MANCOVA results and standardized canonical coefficients for stored glycogen, protein and lipid content in male and female *Aedes aegypti* mosquitoes. Significant effects in MANCOVA are highlighted in boldface. For significant effects, standardised canonical coefficients for the first canonical variate are included; the magnitude of standardised canonical coefficients indicate the magnitude of the variable’s contribution to the significant effects in MANCOVA (Scheiner, 1993).

**Fig 3:**
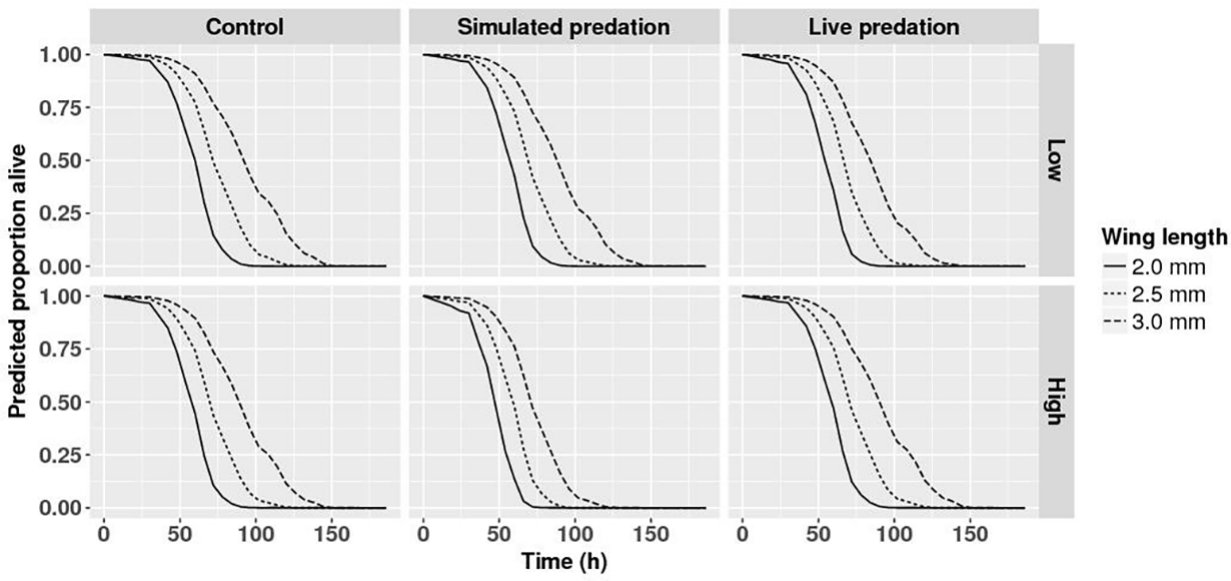
Survival analysis for female mosquitoes. Survival analysis describing predicted survivorship of *Aedes aegypti* for intraspecific competition × predation treatments across replicate containers for female mosquitoes exposed to ‘medium’ nutrition treatment. Panels along the Y and X axes represent intraspecific competition and predation treatments respectively. Data were statistically tested using Cox proportional hazard regression and significant effects are described in Table 4.

Wing length was strongly and positively related to adult longevity (Table 4) with a hazard ratio <1.0 (Table 4) indicating that hazard of death decreased significantly per mm increase of wing length. Nutrition and the interaction of nutrition and competition significantly impacted adult female longevity; the hazard of death decreased significantly with decreasing intraspecific competition in ‘low’ and ‘high’ nutrition treatments. Female longevity was further significantly affected by competition, and two-way interactions of nutrition or competition and predation threat (Fig 3 and Table 4). The hazard of death decreased significantly with an increase in larval nutrition across all three predator treatments. While the hazard of death for females developing in nutrition treatments ‘low’ and ‘medium’ are both greater than that in ‘high’, and not different from each other across the three predator treatments, the magnitudes of the differences are not consistent. In controls, the differences are larger and the difference in hazard of death between nutrition treatments ‘medium’ and ‘high’ is relatively small when compared to the other two predator treatments. No effect of intraspecific competition on hazard of death was observed in females exposed to live predator treatment (Fig 3). These observed effects of manipulated variables on longevity of adult females went beyond the significant effect of wing length.

**Table 4:** Survival analysis.

Source	MALE				FEMALE			
	χ^2^	*df*	*P*	Hazard ratio	χ^2^	*df*	*P*	Hazard ratio
Wing length (WL)	**41.2691**	**1**	**<0.0001**	**0.011**	**48.0419**	**1**	**<0.0001**	**0.136**
Competition	**9.8957**	**1**	**0.0017**		**17.5327**	**1**	**<0.0001**	
Nutrition	1.7036	2	0.4266		**85.4208**	**2**	**<0.0001**	
Predation	3.4656	2	0.1768		3.2561	2	0.1963	
Comp. × Nut.	**7.7694**	**2**	**0.0206**		**7.2452**	**2**	**0.0267**	
Comp. × Pred.	4.3354	2	0.1144		**9.5702**	**2**	**0.0084**	
Nut. × Pred.	10.5986	4	0.0315		**9.7766**	**4**	**0.0444**	
Comp. × Nut. × Pred.	3.9103	4	0.4183		3.3134	4	0.5068	
WL × Comp.	**8.5251**	**1**	**0.0035**					
WL × Nut.	0.4501	2	0.7985					
WL × Pred.	4.2570	2	0.1190					
WL × Comp. × Nut.	**6.5910**	**2**	**0.0370**					
WL × Comp. × Pred.	4.8184	2	0.0899					
WL × Nut. × Pred.	**10.5450**	**4**	**0.0322**					
WL × Comp. × Nut. × Pred.	4.0026	4	0.4056					

Results for the Cox proportional hazards regression analysis of survival data of male and female *Aedes aegypti* mosquitoes. Significant main effects and interactions (significant at α = 0.05) are indicated in boldface. Pairwise contrasts between treatment categories are not included for brevity. For the continuous variable wing length, the hazard ratio is the ratio of hazard rate for a 1 mm increase of wing length. The hazard ratio below 1.0 indicates hazard declines with wing length. Because of significant differences among treatment combinations in the relationship of longevity to wing length, the wing length hazard ratio for males is merely an overall average that hides much variation.

### Males

The effects of manipulated variables on development time and adult body mass of males were similar to those for females. However, the effect of treatments, in general, were relatively more pronounced on females than males; particularly, the effect of treatments on eclosion time of males was small when compared to that on females (Fig 2 and refer SCCs in Table 2). Impact of larval competition (SCCs: Male, -0.2550; Female, -0.6267) was relatively greater for development time of females compared to males. Similarly, the effect of nutrition availability was greater for development time for females than for males (SCCs: Male, -0.2033; Female, -0.4407 & 1.2982). Effects of predation and all its interactions were not statistically significant (Table 2).

**Fig 4:**
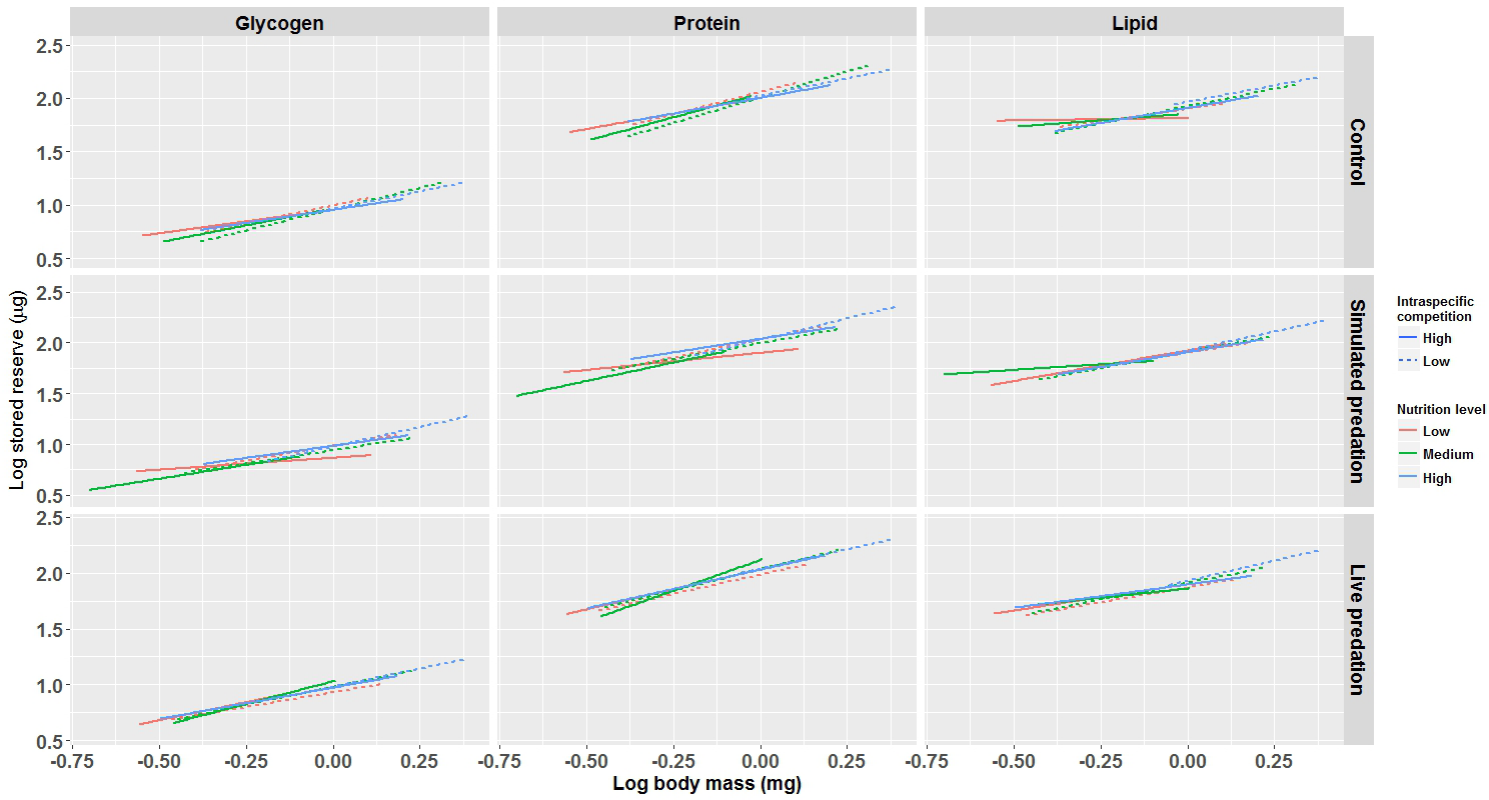
Stored teneral reserves in male mosquitoes. Predicted lines for log (stored teneral reserves–glycogen, protein and lipid) ~ log (adult body mass) for larval nutrition × intraspecific competition × predation treatments across replicate containers for male mosquitoes. All variables are log transformed. Data were statistically tested using a mixed model MANCOVA and significant effects are described in Table 3.

Body mass of males, as was true for females, had a strong positive relationship to teneral reserve components. In contrast to females, the interaction of nutrition and predation treatments, and interactions between each of the main effects and body mass had significant impacts on reserve components in males (Fig 4 and Table 3). This suggests that the slopes for the relationships of glycogen, protein, and lipid to adult body mass were different for males in each of the treatment groups (SCCs in Table 3). In Fig 4, several of the panels show that for combinations of high competition and low or medium nutrition the slope of the reserve vs. body mass relationship is conspicuously shallower than those for other combinations (e.g., control-lipid panel).

**Fig 5:**
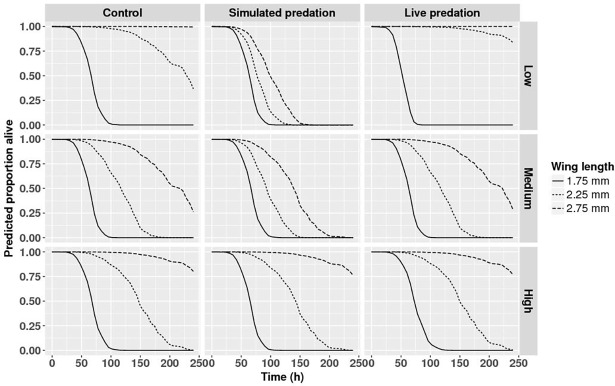
Survival analysis for male mosquitoes. Survival analysis describing predicted survivorship of *Aedes aegypti* for wing length × larval nutrition × predation treatments across replicate containers for male mosquitoes subjected to ‘high’ intraspecific competition. Panels along the Y and X axes represent nutrition and predation treatments respectively. Data were statistically tested using Cox proportional hazard regression and significant effects are described in Table 4.

The strong positive relationship between wing length and adult longevity was present for male mosquitoes, and as with females the hazard of death decreased significantly per mm increase of wing length. This effect of size on male longevity differed significantly among the 18 different treatment combinations (Table 4, interactions with wing length) and generally (15/18 cases) decreased with size more rapidly than that for females (i.e., the hazard ratio is farther 1.0, Table 4). The best estimate of the overall average wing length hazard ratio for males is given in Table 4, but this value obscures the significant variation among treatment groups. Predation treatments interacted with nutrition treatments and wing length to affect male longevity (Table 4, Fig 5). With high nutrition, male size-dependent survival curves were similar for all predation treatments (Fig 5, High), but with medium and low nutrition, these curves differed among predation treatments, with simulated predation producing the steepest size-dependent survival curves (Fig 5, Low, Medium).

## Discussion

Our results suggest that interactions between non-lethal effects of predation, larval nutrition and intraspecific competition reduced longevity of adult females and altered the biochemical composition of stored nutrients in adult males. Specifically, the interaction between the three manipulated variables decreased stored glycogen and increased protein and lipid contents in male mosquitoes. No marked differences were observed between simulated and live predation treatments. Mosquito larvae exposed to reduced nutrition levels and elevated intraspecific competition had slowed larval development. They eclosed as smaller adult mosquitoes with reduced body mass and shorter wing lengths. Longer-winged mosquitoes lived longer and lighter individuals contained reduced levels of protein, glycogen and lipid. Male and female mosquitoes responded differently to the conditions they encountered as larvae. The quality of larval environment affected the development and adult characteristics of females more severely than those of males.

Studies on larvae of mosquitoes show that survival to adulthood, time to adulthood, and adult body size are food and density-dependent [39,65,80]. Results from this study are consistent with those findings. Larvae took longer to metamorphose with reduced per capita food availability, which, in turn, depended on the initial amount of food added to experimental containers (i.e., nutrition treatments) and density of larvae (Table 2). Food levels across treatments were not likely a limiting factor early in development and larvae progressed through 1^st^ and 2^nd^ instars synchronously. As larvae consumed the available food, resource depletion gradually intensified competition for food. At low larval densities, different levels of nutrition availability influenced time to metamorphosis but not survival to adulthood (Fig 1 and Table 1). Nutrition treatments ‘low’ and ‘medium’ with high density of larvae had reduced per capita food availability thus resulting in earlier onset of nutrition limitation. Few larvae in these nutrition treatments managed to survive to adulthood (Fig 1 and Table 1), with most dying between 2^nd^ and 4^th^ instars (K.C., personal observations during the study). Density-dependent competition did not influence survival in high-nutrition treatments as enough food was available for the developing larvae.

Much of the nutrition assimilated in early stages of larval development is allocated towards structural growth [81]. For metamorphosing insects, such allocations are crucial as they need to surpass certain physiological checkpoints to progress through each stage of development [74]. Though little is known about these checkpoints, they seem to be influenced by a suite of factors like growth rate, nutritional status of developing larvae, body size, temperature, and noticeably sex of the individual. Male larvae develop and emerge ahead of female larvae—this gender-specific difference in development rate (protandry), occurs in several insects and other arthropods where females are monogamous [82]. Theories on sexual and natural selection predict differential adaptive benefits of protandry for both males and females; males benefit by mating multiple times and females by enhanced fecundity [68,73]. Previous studies have shown sex-specific responses to biological interactions in protandrous insects [75,83,84]. Development time for males was not strongly affected by nutrition and density, but adult size and size-dependent body composition changed dramatically as evident in larvae exposed to ‘low’ and ‘medium’ nutrition and ‘high’ intraspecific competition (Figs 2 and 4). In contrast female development time and body size both changed dramatically in response to nutrition and density (Fig 2), but female body composition was very consistently related to adult size independent of any manipulated variable (Table 3). Being exposed longer to their larval habitats, females bear the brunt of unfavorable conditions relatively more and on average suffer higher cumulative larval mortality than males. These sex-specific effects could account for the male-biased sex ratios observed in mosquito populations in the wild [85].

Analyzing the way developing males and females utilize assimilated nutrition is a good representation of how life history characteristics are shaped by larval conditions in both the sexes. Body size of adult mosquitoes at emergence increased with food availability in the larval environment; the magnitude of increase, however, was negatively correlated with the level of larval competition. In mosquitoes, females are almost always larger as adults than males [86]. Adult males had teneral reserve masses comparable to those in females that are larger in size (compare nutrient reserves in males and females at any body size). For females, none of the manipulated variables affected teneral reserve composition relative to body size. In contrast for males, the relationship between teneral reserve components and body size depended heavily on the treatment combinations they were exposed to as larvae (Table 3). Of the three reserve components, lipid is most responsive to larval environmental conditions, followed by protein and glycogen (SCCs). Lipid reserves are indispensable in structural growth, regulating metabolism and reproduction [87,88]. Quantity of lipid in an individual also indicates the overall quality of the mosquito’s nutrition reserve. Protein being a structural component contributes to overall development, increase in biomass and production of seminal fluids [89]. Glycogen reserves are essential for flight activity [90].

The relationship between adult body size, nutrient reserves and fitness in mosquitoes is well known [62]. Larger male mosquitoes are successful at acquiring mates early, transfer more sperm during mating and therefore achieve greater reproductive success [91,92]. Larger female mosquitoes are better at seeking hosts and using blood meals for production of a clutch of eggs [93–95]. This largely minimizes the use of teneral reserves for reproductive needs which otherwise could be used by female mosquitoes in oviposition site selection, immune function, etc. [96–98]. Moreover, females are efficient in supplementing their teneral reserves with nutrients extracted from blood meals [99]. Also, adult mosquitoes supplement the need for more reserves via other mechanisms–glycogenesis and lipogenesis from carbohydrate sources. The ability to perform these syntheses has a strong positive correlation with body size [100].

Likewise, adult longevity in mosquitoes is positively correlated with amount and quality of nutritional reserves and body size [66]. Living longer enables male and female mosquitoes to have multiple reproductive cycles and increases their potential lifetime fitness. Likelihood of females becoming vector competent increases with age. Greater longevity therefore enhances vectorial capacity of the population, although smaller females may in some cases be more susceptible to infection given exposure [68,101–103]. While nutrient reserves and longevity scaled consistently with body size in females, the scaling relationship between these dependent variables and body size differed with treatment combination in males (Figs 3 and 5 and Tables 3 and 4). Thus, because the larval environment impacts body size and nutrient reserves, the larval environment indirectly determines potential for individuals and populations to transmit pathogens, in addition to affecting fitness of resulting adults.

Main effects of predation threat on ontogeny and lifehistory traits of *A*. *aegypti* were not significant. Predation threat had significant interactive effects on adult female longevity and adult male body composition and size-dependent survival, suggesting that non-lethal effects of predation are dependent on the environmental context in which larvae develop, and are manifest in adult traits. Predation threat impacts adults to a greater extent when larval food is scarce and larval densities are high. A few other studies investigating predator-prey interactions in *Aedes albopictus*, *Aedes triseriatus* and *Aedes notoscriptus* have reported a direct effect of predation threat on larval survivorship, time to metamorphosis, body size and starvation resistance of adult mosquitoes [43,62,104,105]. Most *Aedes* larvae inhabit small ephemeral aquatic habitats such as tree holes and man-made containers, and they need to make the most out of the available conditions to survive, metamorphose and escape the uncertainties associated with their habitat. As anti-predatory responses, such as altered behavior, are costly, selection would favor larvae that stringently assess the habitat using available chemical, visual and tactile cues to predation risk and respond only when benefits of responses outweigh costs [106,107]. In this experiment, the *Aedes aegypti* larvae could detect the caged *Toxorhynchites* larva chemically, visually, and perhaps tactilely (via water currents) but could not directly encounter the predator. However, the larvae were free to contact the physical remnants of predation (e.g., predator faeces, victim corpses). Previous work suggests that *Aedes* larvae respond most strongly to solid residues of predation [60,108]. Direct predator encounters may elicit a stronger response in *A*. *aegypti*, but it is impossible to assess the non-consumptive effects of predation if freely swimming *Toxorhynchites* are allowed to have lethal effects on prey.

In summary, the observed effects of non-lethal predation threat are subtle and context dependent on food and intraspecific larval density. The observed predator effects impinge on adult body composition (in males) and adult longevity (in females and males), rather than on the more obvious outcomes of larval development, such as adult size, development time, and survivorship to adulthood. The effects on female longevity are particularly intriguing as longevity is directly linked to demography and vectorial capacity. The findings from this study show clearly how the environments encountered by juveniles can have long-term effects on adult biology. Such effects are likely to be important for basic understanding of population and community-level consequences of interactions between organisms and their environment, and, for mosquitoes, applied understanding of how larval environments impact the disease-transmitting stage.
